# A298 HIGH MORTALITY NOTED AMONG PATIENTS ADMITTED WITH ALCOHOL ASSOCIATED HEPATITIS: A DESCRIPTIVE STUDY

**DOI:** 10.1093/jcag/gwad061.298

**Published:** 2024-02-14

**Authors:** G Malhi, C McChesney, D Hudson, E Stephenson, Y Almahanna, J Arab

**Affiliations:** Gastroenterology, Western University, London, ON, Canada; Gastroenterology, Western University, London, ON, Canada; Gastroenterology, Western University, London, ON, Canada; Gastroenterology, Western University, London, ON, Canada; Gastroenterology, Western University, London, ON, Canada; Gastroenterology, Western University, London, ON, Canada

## Abstract

**Background:**

Alcohol associated hepatitis is a syndrome related to alcohol use disorder characterized by jaundice, malaise, decompensated liver disease, and coagulopathy. This is associated with bacterial infections, the development of chronic liver failure, and high short-term mortality. However, the rates at which these events occur is less understood, with a broad range of estimates reported.

**Aims:**

To understand the demographic profile of those admitted with alcohol associated hepatitis assess its impact on resource utilization, complications in hospital, and mortality.

**Methods:**

This study examined a subset of individuals with alcohol associated hepatitis who were admitted to the GI and Medicine Wards between 2010 and 2023.

**Results:**

70 encounters were identified and interpreted. The average age at admission was 48.3 ± 11.4 years old. 62.8% were male and 22% reported cannabis use. 54% had a previous reported history of alcohol use disorder. The average MELD-Na score at the time of admission was 25.9 ± 7.7.

The Median length of stay was 8.5 days (IQR 4.5,15). 63% were provided steroids in hospital and 33.3% developed infectious complications. 36.3% of patients had a GI bleed reported during admission. 4% of patients underwent a transplantation during follow-up. Approximately 44.3% experienced mortality with a 30-day mortality rate found to be 28.6%.

**Conclusions:**

Alcohol associated hepatitis patients are prone to developing infections and bleeding during admission. This diagnosis also carries a high mortality rate, particularly within the first 30 days. Further studies examining the global impact of alcohol associated hepatitis should be considered.

Table 1: Baseline Demographics

**Funding Agencies:**

None

**Table:** 

Parameter	Number (%)
**Total**	70 (100)
**Sex**	
Male	44 (62.9)
Female	26 (37.1)
**Age**	
Mean	48.3 ± 11.4
Range	23-72
**Previously Diagnosed Cirrhosis**	
Yes	27 (38.6)
No	43 (61.4)
**Marijuana Use**	
Yes	16 (22.9)
No	54 (77.1)

